# PD-1 Alleviates Cisplatin-Induced Muscle Atrophy by Regulating Inflammation and Oxidative Stress

**DOI:** 10.3390/antiox11091839

**Published:** 2022-09-18

**Authors:** Xiaoguang Liu, Miaomiao Xu, Yang Yu, Yingjie Chen, Xinyu Weng, Lin Zhu

**Affiliations:** 1 School of Sport and Health, Guangzhou Sport University, Guangzhou 510500, China; 2 Guangdong Provincial Key Laboratory of Physical Activity and Health Promotion, Guangzhou Sport University, Guangzhou 510500, China; 3 School of Kinesiology, Shanghai University of Sport, Shanghai 200438, China; 4 Department of Physiology and Biophysics, University of Mississippi Medical Center, Jackson, MS 39216, USA; 5 Department of Cardiology, Zhongshan Hospital, Shanghai Institute of Cardiovascular Diseases, Fudan University, Shanghai 200437, China

**Keywords:** skeletal muscle, atrophy, inflammation, oxidative stress

## Abstract

Skeletal muscle atrophy is an important characteristic of cachexia, which can be induced by chemotherapy and significantly contributes to functional muscle impairment. Inflammation and oxidative stress are believed to play important roles in the muscle atrophy observed in cachexia, but whether programmed cell death protein 1 (PD-1) is affected by this condition remains unclear. PD-1 is a membrane protein that is expressed on the surface of many immune cells and plays an important role in adaptive immune responses and autoimmunity. Thus, we investigated the role and underlying mechanism of PD-1 in cisplatin-induced muscle atrophy in mice. We found that PD-1 knockout dramatically contributed to skeletal muscle atrophy. Mechanistically, we found that E3 ubiquitin-protein ligases were significantly increased in PD-1 knockout mice after cisplatin treatment. In addition, we found that PD-1 knockout significantly exacerbated cisplatin-induced skeletal muscle inflammation and oxidative stress. Moreover, we found that there were significant increases in ferroptosis-related and autophagy-related genes in PD-1 knockout mice after cisplatin treatment. These data indicate that PD-1 plays an important role in cisplatin-induced skeletal muscle atrophy.

## 1. Introduction

Skeletal muscle is one of the most important organs found in humans and is fundamental for exercise and respiration [1]. The loss of muscle mass is not only a hallmark of sarcopenia, but also a common consequence of cancer, chronic heart failure, lung disease, renal disease and chemotherapy administration [2]. In addition, the loss of skeletal muscle mass impairs exercise performance, reduces quality of life and increases mortality [3]. Chemotherapy is one of the most successful treatments for cancer patients. Cisplatin is a first-line therapy that is widely used to treat several types of cancer, including Ewing sarcoma, rhabdomyosarcoma and osteosarcoma. However, cisplatin-based therapy is associated with significant side effects, and skeletal muscle wasting is the most relevant [2,4,5]. Attenuating cisplatin-induced skeletal muscle atrophy is an important approach to identifying novel promising therapeutic targets. To date, the mechanism of cisplatin-induced skeletal muscle atrophy has not been fully elucidated.

Cisplatin-based chemotherapy increases the recruitment of inflammatory cells and the production of inflammatory cytokines [4,5]. Programmed cell death protein 1 (PD-1) is a membrane protein that is expressed on the surface of activated T cells [6,7]. PD-1 plays an important role in adaptive immune responses and autoimmunity [8]. In addition, inhibition by PD-1 and its ligands has been demonstrated to be successful in treating various cancers [9,10,11]. However, immunotherapies targeting PD-1 and its ligand PD-L1 result in various organ and tissue toxicities [12,13,14]. In a recent study, we found that the pharmacological and genetic inhibition of PD-1 impaired muscle angiogenesis and contributed to muscle atrophy after hindlimb ischemia [15]. In addition, the inhibition of PD-1 or its ligands by anti-PD-1 or PD-L1 inhibitors resulted in muscle weakness, asthenia, and myasthenic-like syndrome [16]. However, the mechanisms of tissue toxicity induced by PD-1 inhibition are not well known.

Since PD-1 modulates inflammatory responses to physiological and pathological stress, we hypothesize that PD-1 may play an important role in muscle atrophy by exacerbating skeletal muscle inflammation. Consequently, we determined the role and mechanism of PD-1 inhibition via genetic PD-1 deletion in mice with cisplatin-induced muscle atrophy and inflammation.

## 2. Materials and Methods

### 2.1. Animals

The C57BL/6 mice were obtained from Guangdong Medical Laboratory Animal Center. The PD-1-KO mice (C57BL/6J background) were purchased from Jackson Laboratory. Male mice at the age of 8 weeks were used for the study and were randomly assigned to different experimental groups. Euthanasia was performed by exsanguination after anesthesia with isoflurane. This investigation conforms with the Guide for the Care and Use of Laboratory Animals published by the US National Institutes of Health (NIH Publication No. 85-23, revised 1996).

### 2.2. Cisplatin-Induced Skeletal Muscle Atrophy

The WT and PD-1-KO mice were administered cisplatin daily (2.5 mg/kg) for 5 days on Days 1–5 and Days 20–25 for a total of 10 times by intraperitoneal (i.p.) injection [4]. No deaths were recorded in any group after this moderate-dose treatment.

### 2.3. RNA Extraction and Gene Expression Analysis

Total RNA was extracted from the gastrocnemius muscle tissue using the TRIzol-chloroform method (Invitrogen, California, USA), according to the manufacturer’s protocol.

Total RNA (1 µg) was used for the first-strand cDNA synthesis with a HiScript II 1st Strand cDNA Synthesis Kit (Vazyme, Nanjing, China). For the quantitative real-time PCR (RT–qPCR), 1 µL of cDNA was amplified using a ChamQ SYBR qPCR Master Mix (Vazyme, Nanjing, China). The relative mRNA expression was normalized to the housekeeping gene glyceraldehyde 3-phosphate dehydrogenase (GAPDH) and was calculated using the 2^−ΔΔCT^ method [17]. All primers used in this study are listed in Table 1.

### 2.4. Histological Analysis

The tibialis anterior (TA) muscle tissue from WT and PD-1-KO mice was fixed in 4% paraformaldehyde in phosphate-buffered saline (Servicebio, Wuhan, China), embedded in paraffin, and cut into 5 μm thick sections. Hematoxylin and eosin (H&E) staining was used to examine the muscle fiber diameters and the cross-sectional areas (CSA). The fiber CSAs and diameters were digitized and analyzed using Image Pro Plus 6 (IPP6) software by manually drawing irregular lines across the muscle fibers. The lengths of the lines and areas (in pixels) generated by IPP6 were converted into micrometers (μm) and square micrometers (μm^2^) using the image scale bars. Approximately 200 fibers from each mouse were measured.

### 2.5. Western Blot Analysis

A chilled radioimmunoprecipitation assay (RIPA) tissue lysis buffer with 1× protease inhibitor cocktail (Sangon Biotech, Shanghai, China) was used to extract total proteins from the skeletal muscle. After incubation on ice for 30 min and centrifugation for 15 min, the proteins were extracted. Bicinchoninic acid kits (Sangon Biotech, Shanghai, China) were used to determine the protein concentration of each supernatant sample. Proteins were separated using 10% SDS–PAGE and transferred to a polyvinylidene fluoride (PVDF) membrane (Millipore Corp., Bedford, MA, USA). The PVDF membrane was blocked with 5% skimmed milk in Tris-buffered saline, containing 0.1% Tween 20, for 1 h. After being blocked, the immunoblots were probed overnight with primary antibodies against 4 hydroxynonenal (Abcam, ab46545), 3-nitrotyrosine (Abcam, ab61392), Fbxo32 (ABclonal, A3193), TLR4 (Servicebio, GB11519), ACSL-4 (Santa Cruz, sc-365230) and beta-actin (Proteintech, 66009-1-Ig) (Table 2). Then, the PVDF membrane was incubated with horseradish peroxidase-conjugated IgG secondary antibodies (Table 3) for 1 h at room temperature. Electrogenerated chemiluminescence kits were used to detect the signal. The staining intensity of the membrane was measured using ImageJ software.

### 2.6. Statistical Analysis

All data are presented as the mean ± standard error. The two groups were compared via Student’s *t*-test using GraphPad Prism 8 software. A two-way ANOVA, followed by a Bonferroni correction post hoc test, was used to test for differences among more than 2 groups. The null hypothesis was rejected at *p* < 0.05.

## 3. Results

### 3.1. Increased PD-1/PD-L1 Expression after Cisplatin Treatment in Wild-Type Mice

**Figure 1:**
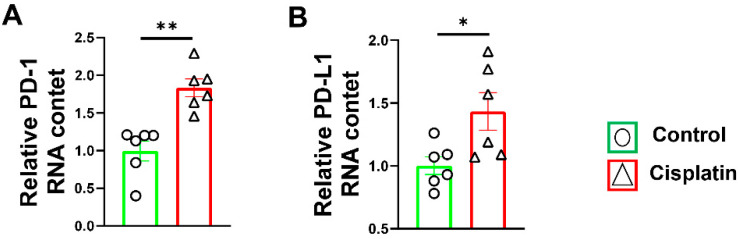
Cisplatin treatment contributed to the expression of PD-1 and PD-L1 in the gastrocnemius muscle. (**A**) The relative mRNA levels of PD-1 in skeletal muscle. (**B**) The relative mRNA levels of PD-L1 in skeletal muscle. mRNAs were normalized to glyceraldehyde-3-phosphate dehydrogenase (GAPDH). *n* = 6 per group. * *p* < 0.05; ** *p* < 0.01; all values are mean ± SEM.

The RT–PCR results showed that PD-1 mRNA expression significantly increased in the skeletal muscle in the cisplatin treatment mice (*p* < 0.01) (Figure 1A). Consistent with the expression of PD-1 mRNA, the expression of PD-L1 mRNA in the skeletal muscle was also increased significantly in the cisplatin-induced muscle atrophy wild-type mice (*p* < 0.05) (Figure 1B).

### 3.2. PD-1^−/−^ Contributed to Skeletal Muscle Atrophy after Cisplatin Treatment

**Figure 2:**
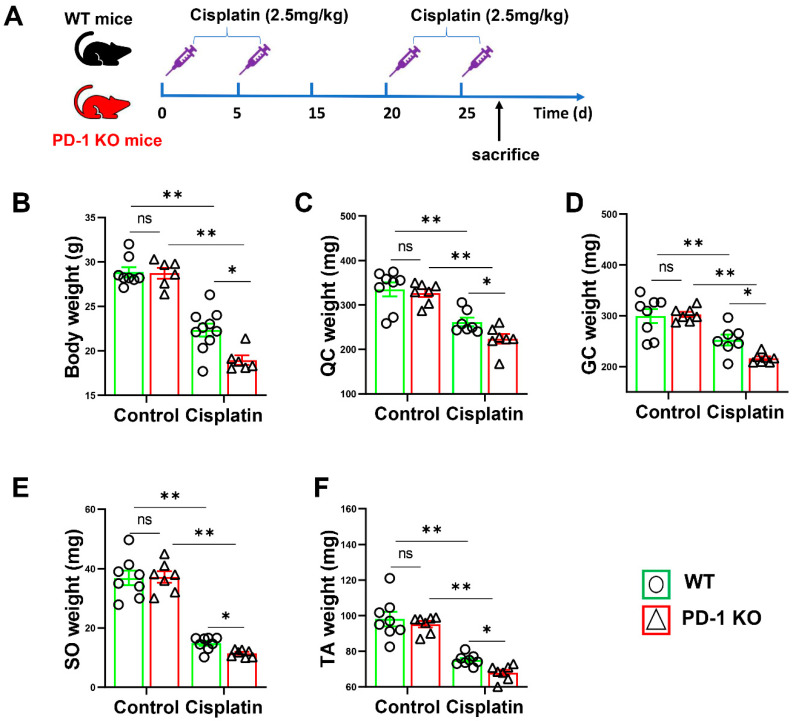
PD-1^−/−^ contributed mice cachexia after cisplatin treatment. (**A**) The diagram shows the time of relevant interventions. (**B**) The body weight of WT and PD-1^-/-^ mice. (**C**) The weight of quadriceps (QC) muscle. (**D**) The weight of gastrocnemius (GC) muscle. (**E**) The weight of soleus (SO) muscle. (**F**) The weight of tibialis anterior (TA) muscle. *n* = 6–9 per group. * *p* < 0.05; ** *p* < 0.01; ns: non-significant. all values are mean ± SEM.

To examine the role of PD-1 in the development of skeletal muscle atrophy, PD-1 knockout (KO) and wild-type (WT) mice were used, as shown in Figure 2A. As shown in Figure 2, the PD-1-KO mice did not show any major changes in total body weight, quadriceps femoris (QC), gastrocnemius muscle (GC), soleus (SO), or tibialis anterior (TA) weight. Cisplatin-induced low body weight and muscle weight were found in both PD-1-KO mice and WT mice. However, the body weight was significantly lower in the PD-1^−/−^ group after cisplatin treatment than in the control group (*p* < 0.01) (Figure 2B). Then, we evaluated the effect of cisplatin on skeletal muscle weight. The results showed that the weights of the QC, GC, SO, and TA were significantly lower in the PD-1^−/−^ mice than in the WT mice after cisplatin treatment (Figure 2C–F).

**Figure 3:**
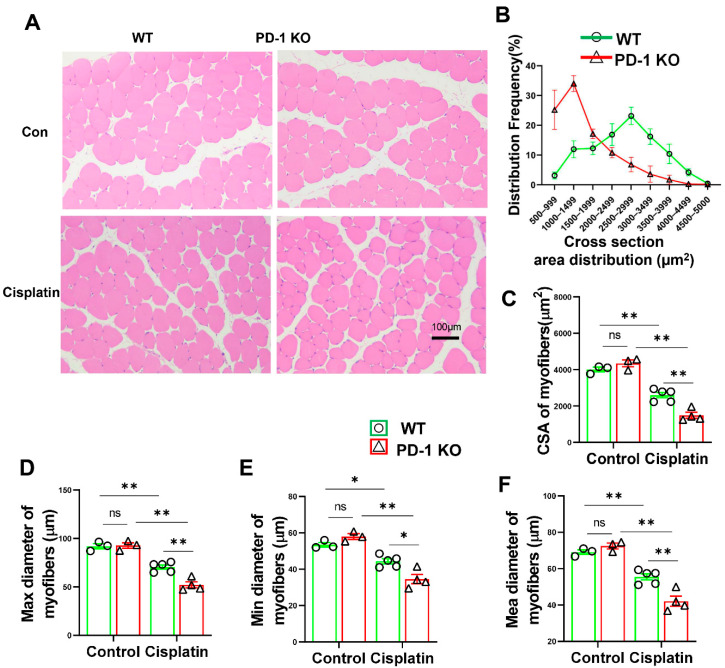
PD-1^−/−^ contributed to muscle atrophy after cisplatin treatment. (**A**) Representative images of H&E staining of skeletal muscle in WT and PD-1^−/−^ mice after cisplatin treatment. (**B**) Percentage distribution of muscle fiber cross-section area (CSA) derived from WT and PD-1^−/−^ mice. (**C**) Quantified myocyte fiber cross-sectional area of WT and PD-1^−/−^ mice after cisplatin treatment. (**D**) Quantified myocyte fiber max diameter of WT and PD-1^−/−^ mice after cisplatin treatment. (**E**) Quantified myocyte fiber minimum diameter of WT and PD-1^−/−^ mice after cisplatin treatment. (**F**) Quantified myocyte fiber means diameter of WT and PD-1^−/−^ mice after cisplatin treatment. *n* = 4–5 per group. * *p* < 0.05; ** *p* < 0.01; ns: non-significant. all values are mean ± SEM.

In order to further confirm that PD-1 KO contributes to muscle atrophy, we analyzed the CSA, maximum diameter, minimum diameter and average diameter of the myofibers. The results showed that the CSA, maximum diameter, minimum diameter and mean diameter of the myofibers were significantly lower in the PD-1^−/−^ mice than in the WT mice after cisplatin treatment (Figure 3), indicating that PD-1^−/−^ contributed to muscle atrophy.

### 3.3. PD-1^−/−^ Enhanced the Expression of E3 Ubiquitin-Protein Ligases after Cisplatin Treatment

**Figure 4:**
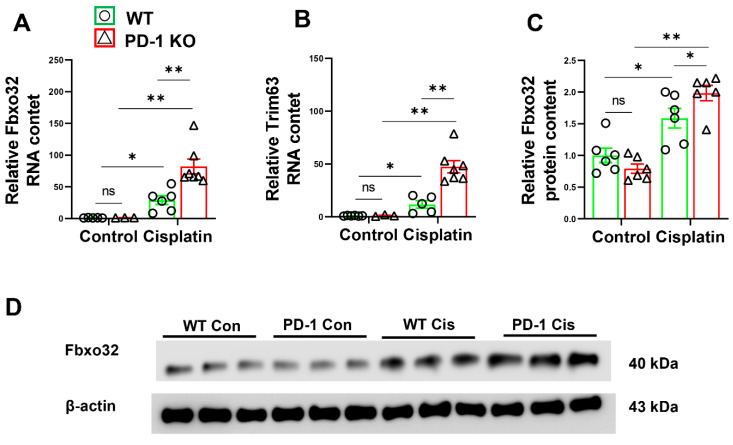
PD-1^−/−^ contributed to the expression of E3 ubiquitin-protein ligases after cisplatin treatment. (**A**) The relative mRNA levels of Fbxo32 in skeletal muscle. (**B**) The relative mRNA levels of Trim63 in skeletal muscle. (**C**) The relative protein levels of Fbxo32 in skeletal muscle. (**D**) The representative protein bands of Fbxo32 and β-actin in skeletal muscle. mRNAs were normalized to glyceraldehyde-3-phosphate dehydrogenase (GAPDH).Proteins were normalized to β-actin. *n* = 5–6 per group. * *p* < 0.05; ** *p* < 0.01; ns: non-significant. all values are mean ± SEM.

Muscle atrophy causes apparent muscle degradation, and the tripartite motif containing 63 (Trim63) and F-box only protein 32 (Fbxo32) are the most intensively studied E3 ubiquitin-protein ligases and are upregulated in atrophy models [18,19]. Therefore, we measured the expression of MuRF1 and Fbxo32 in the skeletal muscle. MuRF1 and Fbxo32 mRNA levels in the normal skeletal muscles were similar in the PD-1^−/−^ and WT mice. However, the expression of MuRF1 and Fbxo32 was significantly increased in the PD-1^−/−^ mice compared with the WT mice after cisplatin treatment (*p* < 0.01) (Figure 4A,B). We further determined the protein level of Fbxo32 in the WT and PD-1^−/−^ mice before and after cisplatin treatment. The results showed that muscle Fbxo32 protein levels were comparable between the PD1^−/−^ mice and WT mice before cisplatin treatment, while the Fbxo32 protein level was significantly higher in the PD-1^−/−^ mice compared with the WT mice after cisplatin treatment (Figure 4C,D).

### 3.4. PD-1^−/−^ Exacerbated Muscle Inflammation in Mice after Cisplatin Treatment

**Figure 5:**
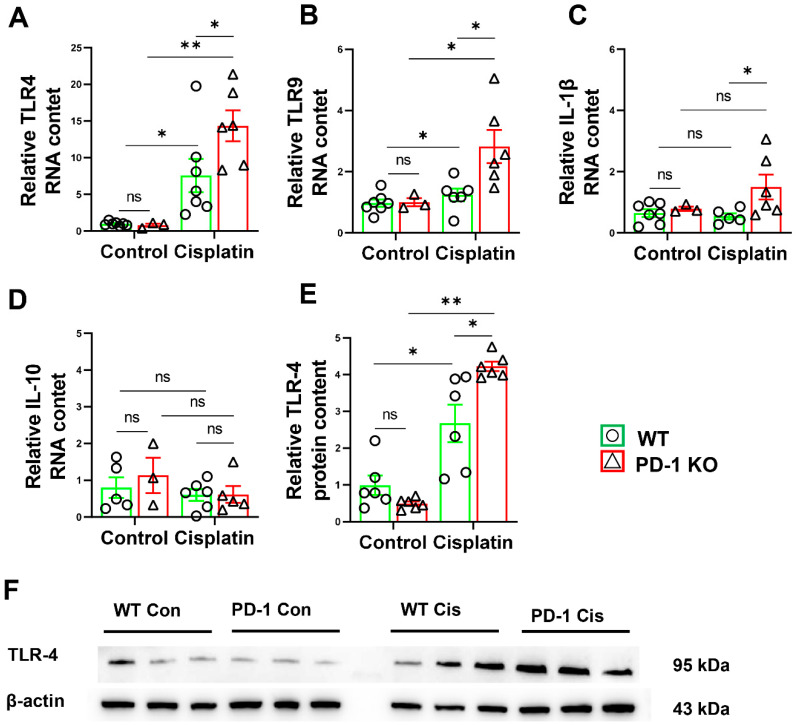
PD-1^−/−^ exacerbated muscle inflammation in mice after cisplatin treatment. (**A**) The relative mRNA levels of TLR4 in skeletal muscle. (**B**) The relative mRNA levels of TLR9 in skeletal muscle. (**C**) The relative mRNA levels of IL-1β in skeletal muscle. (**D**) The relative mRNA levels of IL-10 in skeletal muscle. (**E**) The relative protein levels of TLR4 in skeletal muscle. (**F**) The representative protein bands of TLR4 and β-actin in skeletal muscle. mRNAs were normalized to GAPDH. Proteins were normalized to β-actin. *n* = 5–6 per group. * *p* < 0.05; ** *p* < 0.01; ns: non-significant. all values are mean ± SEM.

Since chemotherapy triggers proteolysis and the release of inflammatory mediators in skeletal muscle [20], we hypothesized that PD-1 could attenuate cisplatin-induced atrophy by exacerbating skeletal muscle inflammation. Consequently, we detected the muscle inflammation in the PD-1^−/−^ mice and WT mice after cisplatin treatment. The RT–PCR results showed that the expressions of IL-1β, TLR4, and TLR9 mRNA in the skeletal muscles of the PD-1^−/−^ mice were significantly higher than those of the wild-type mice (Figure 5). Consistent with these observations, the protein levels of TLR4 were significantly higher in the PD-1^−/−^ mice than in the WT mice after cisplatin treatment (Figure 5).

### 3.5. PD-1^−/−^ Exacerbated Muscle Oxidative Stress in Mice after Cisplatin Treatment

**Figure 6:**
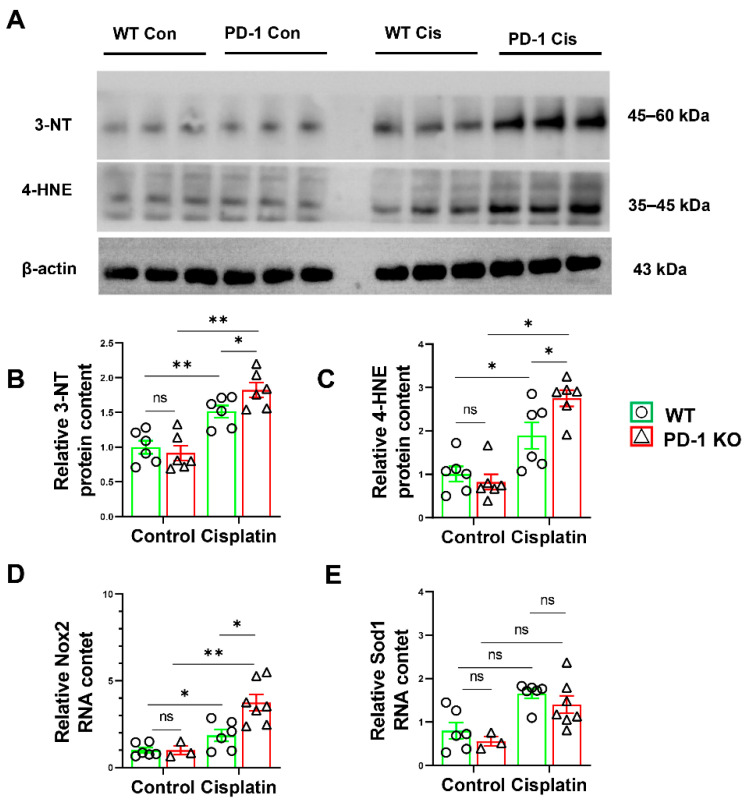
PD-1^−/−^ contributed muscle oxidative stress after cisplatin treatment. (**A**) The representative protein bands of 3-NT, 4-HNE and β-actin in skeletal muscle. (**B**) The relative protein levels of 3-NT in skeletal muscle. (**C**) The relative protein levels of 4-HNE in skeletal muscle. *n* = 6 per group. (**D**) The relative mRNA levels of Nox-2 in skeletal muscle. (**E**) The relative mRNA levels of Sod1 in skeletal muscle. * *p* < 0.05; ** *p* < 0.01; ns: non-significant. all values are mean ± SEM.

Reactive oxidative species (ROS) contribute to oxidative damage in the muscles of cisplatin-treated rodents [21]. We measured the ROS in the skeletal muscle by WB. As expected, cisplatin caused a significant increase in 4-hydroxynonenal (4-HNE) and 3-nitrotyrosine (3-NT) levels; however, 4-HNE and 3-NT were significantly increased in the PD-1^−/−^ mice and WT mice after cisplatin treatment (Figure 6). In addition, the quantitative RT–PCR showed that cisplatin treatment led to significant increases in Nox-2 in the WT and PD-1^−/−^ mice, and these levels were significantly increased in the PD-1^−/−^ mice (Figure 6). Taken together, these data indicate that PD-1^−/−^ exacerbated muscle oxidative stress in the mice after cisplatin treatment.

### 3.6. PD-1^−/−^ Exacerbated the Expression of Ferroptosis-Related Genes in Mice after Cisplatin Treatment

**Figure 7:**
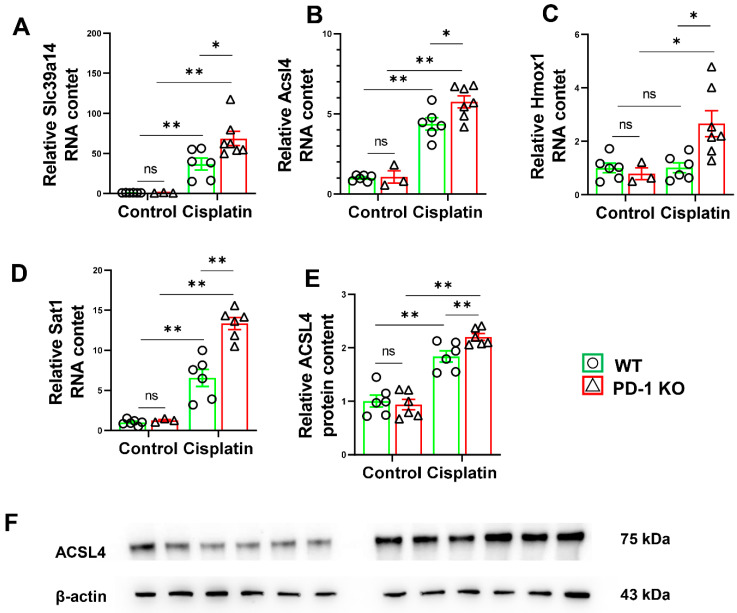
PD-1^−/−^ exacerbated muscle ferroptosis in mice after cisplatin treatment. (**A**) The relative mRNA levels of Slc39a14 in skeletal muscle. (**B**) The relative mRNA levels of Acsl4 in skeletal muscle. (**C**) The relative mRNA levels of Hmox1 in skeletal muscle. (**D**) The relative mRNA levels of Sat1 in skeletal muscle. (**E**) The relative protein levels of Acsl4 in skeletal muscle. (F) The representative protein bands of Acsl4 and β-actin in skeletal muscle. *n* = 5–6 per group. * *p* < 0.05; ** *p* < 0.01; ns: non-significant. all values are mean ± SEM.

Growing evidence suggests that ferroptosis is involved in the pathogenesis of many diseases such as sarcopenia [22]. We further examined ferroptosis-related genes in the cisplatin-treated mice. Acyl-CoA synthetase long-chain family member 4 (ACSL4), heme oxygenase-1 (Hmox1), spermidine/spermine N1-acetyltransferase 1 (Sat1), and transmembrane metal-ion transporter solute carrier family 39 member 14 (SLC39A14) expression levels were significantly increased in the PD-1^−/−^ and WT mice after cisplatin treatment. Interestingly, we found that after cisplatin treatment, compared with the WT mice, the expression levels of ACSL4, Hmox1, Sat1, and SLC39A14 in the PD-1^−/−^ mice increased significantly (Figure 7).

### 3.7. PD-1^−/−^ Exacerbated the Expression of Autophagy-Related Genes in Mice after Cisplatin Treatment

**Figure 8:**
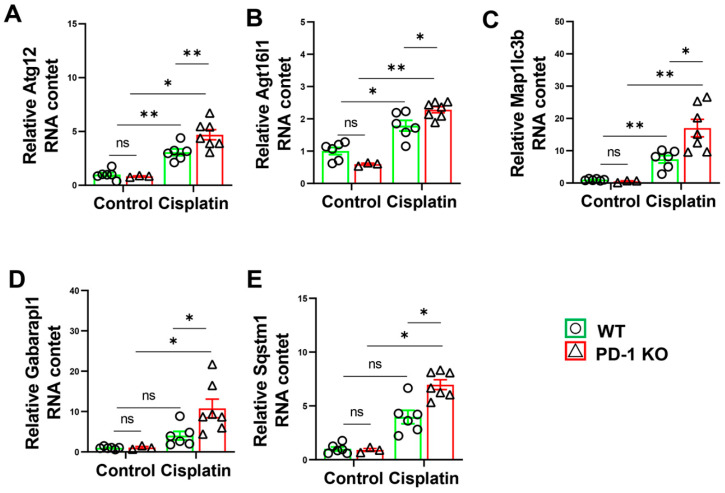
PD-1^−/−^ exacerbated muscle autophagy in mice after cisplatin treatment. (**A**) The relative mRNA levels of Atg12 in skeletal muscle. (**B**) The relative mRNA levels of Atg16l1 in skeletal muscle. (**C**) The relative mRNA levels of Map1lc3b in skeletal muscle. (**D**) The relative mRNA levels of Gabarapl1 in skeletal muscle. (**E**) The relative mRNA levels of Sqstm1 in skeletal muscle. mRNAs were normalized to GAPDH. *n* = 5–6 per group. * *p* < 0.05; ** *p* < 0.01; ns: non-significant. all values are mean ± SEM.

Since skeletal muscle atrophy is characterized by changes in atrophy-related genes, we detected the atrophy-related genes in the PD-1^−/−^ and WT mice before and after cisplatin treatment. The quantitative RT–PCR showed that the levels of Atg12, Atg16L1, Map1lc3b, and Sqstm1 in the PD-1^−/−^ mice were similar to those in the WT mice. However, cisplatin treatment significantly increased the levels of Atg12, Atg16L1, Map1lc3b, and Sqstm1 levels in the WT and PD-1^−/−^ mice, and these levels significantly increased in the PD-1^−/−^ mice (Figure 8).

## 4. Discussion

Skeletal muscle atrophy is a devastating complication of cancer that damages quality of life [23]. Chemotherapeutic drugs such as cisplatin induce muscle atrophy during cancer treatment [5,24]. In this study, we provide the first experimental evidence that genetic PD-1 deficiency contributes to skeletal muscle atrophy in mice after cisplatin treatment. In addition, we provide several lines of evidence that PD-1^−/−^ significantly exacerbated cisplatin-induced inflammation, oxidative stress, ferroptosis and autophagy in the muscle tissue. These findings suggest that PD-1 plays an important role in inhibiting cisplatin-induced skeletal muscle atrophy by regulating muscle inflammation, oxidative stress, ferroptosis and autophagy.

PD-1 is expressed on activated immune cells and regulates ongoing immune responses [25]. As a ’coinhibitory’ receptor, PD-1 can function as a break for the immune response through its ligands PD-L1 and PD-L2 [13]. In a wide range of human cancers, immunotherapies that target the PD-1/PD-L1 axis have displayed extraordinary efficacy [26]. However, some cancer patients treated with anti-PD-1 or anti-PDL1 develop muscle weakness, necrosis, and inflammation [16]. Consistent with these results, we found that PD-1 knockout contributed to cisplatin-induced skeletal muscle atrophy.

Cisplatin,a well-known chemotherapeutic drug, has been used for the treatment of numerous human cancers. PD-1/PD-L1 axis blocking can improve cisplatin’s chemotherapy effect in osteosarcoma in vitro and in vivo [27]. Consistent with the reports that cisplatin treatment significantly increased PD-L1 expression in tumor and cancer cell lines [27,28], we found that PD-1 and PD-L1 are increased in cisplatin-induced skeletal muscle wild-type mice, and may play an important role in the process of muscle atrophy.

Atrogenes are genes that are systematically expressed during the muscle atrophying process [29]. Consistent with the reports that Trim63 and Fbxo32, which are common, intensively studied atrogenes, are upregulated in atrophy mice, we found that muscle Trim63 and Fbxo32 were significantly increased in the PD-1^−/−^ mice muscle compared with the WT mice. The dramatic increase in atrogenes in the PD-1^−/−^ mice after cisplatin-induced skeletal muscle atrophy supports the idea that PD-1 KO contributes to the expression of atrogenes in mice after cisplatin treatment.

Since inflammation regulates cisplatin-induced skeletal muscle atrophy [30,31] and PD-1 plays an important role in regulating inflammation, inflammation was detected in cisplatin-induced skeletal muscle. Consistent with previous reports [32,33], we found that muscle TLR4 and TLR9 levels were significantly increased in the WT and PD-1-KO mice after cisplatin treatment, and skeletal muscle inflammation was more apparent in the PD-1-KO mice than in the WT mice. In addition, our previous study showed that PD-1 alleviated skeletal muscle injury and angiogenesis, in response to muscle contusion and hindlimb ischemia, by regulating inflammatory responses [3,15]. Moreover, a previous study demonstrated that PD-1 was a major protective pathway that limited inflammation in murine experimental stroke [34] and atherosclerotic lesions [35].

Oxidative stress is considered to be an important cause of cisplatin-induced skeletal muscle atrophy [5]. Our previous study showed that PD-1 knockout exacerbated oxidative stress in skeletal muscle after hindlimb ischemia [15]. Moreover, PD-1 knockout was shown to exacerbate myocyte oxidative stress [36]. Consistent with these findings, the present study showed that PD-1 knockout significantly increased 4-HNE and 3-NT levels and exacerbated oxidative stress in skeletal muscle, contributing to cisplatin-induced muscle atrophy. In addition, studies have shown that ferroptosis can be induced by oxidative stress in many pathological states.

Ferroptosis is a newly discovered form of regulated cell death that is characterized by iron-dependent cell death, the presence of smaller mitochondria, mitochondrial cristae, and outer mitochondrial membrane rupture [37]. Chen et al. recently reported that iron overload-induced ferroptosis plays an essential role in sarcopenia [22]. Studies have shown that the expression of the iron metabolism-related gene SLC39A14 (ZIP14) is upregulated in cachectic muscles from rodents and humans with various metastatic cancers [38,39]. Consistent with these studies, our findings showed that the cisplatin-induced mRNA expression of the ferroptosis-related genes ACSL4, Hmox1, Sat1, and SLC39A14 in skeletal muscles was significantly increased by PD-1 knockout, suggesting that PD-1 protects skeletal muscle against ferroptosis.

Muscle atrophy occurs as the result of an imbalance between autophagy activation and inhibition [40]. In various skeletal muscle atrophy models, such as starvation, aging, and cancer, the regulation of autophagy protects against skeletal muscle atrophy. Some researchers have demonstrated the upregulation of sqstm1 expression in aged mouse muscles [41,42]. Patel et al. reported that the mild attenuation of autophagy by Atg16L1 knockout accelerated skeletal muscle atrophy during starvation [43]. Abarapl1 is a marker of autophagy that was shown to increase in the abdominal muscles of cachexia patients compared with healthy controls [44]. Mishra et al. reported that the autophagy-related gene Atg12 was upregulated in skeletal muscle in response to fasting [45]. Consistent with the reports that autophagy-related genes were more highly expressed in wasting muscle, we found that the expression of autophagy-related genes (Atg12, Atg16L1, Map1lc3b, Sqstm1) was significantly increased in the PD-1^−/−^ mice compared with the WT mice after cisplatin treatment.

The present study has several limitations that need to be addressed in future studies. First, although cisplatin-induced skeletal muscle atrophy is a commonly used animal model for various studies, since clinical muscle atrophy often occurs in cancer cachexia, the findings obtained from normal mice may not fully mimic those of clinical patients. Furthermore, we only studied male mice in the present study. However, cisplatin-induced muscle atrophy was observed in both male and female mice, and since PD-1 regulates inflammation and oxidative stress in both male and female mice in many experimental models, we anticipate that PD-1 knockout would have similar impacts on skeletal muscle atrophy in both sexes. In addition, C57BL/6J mice, and not WT-generated backcrossing PD-1 KO mice with C57BL/6J mice, were used in this study. However, the background of the PD-1 KO mice was C57BL/6, and the Jackson Laboratory suggested using the C57BL/6 mice as a control for the PD-1 KO mice.

## 5. Conclusions

The present study demonstrated for the first time that PD-1 plays an important role in regulating cisplatin-induced muscle atrophy by modulating skeletal muscle E3 ubiquitin-protein ligases, inflammation, oxidative stress, ferroptosis and autophagy.

## Figures and Tables

**Table 1:** Primer sequences of RT–qPCR.

Targets	Accession Number	Forward Primer	Reverse Primer
GAPDH	NM_008084	ACTCCACTCACGGCAAATTC	TCTCCATGGTGGTGAAGACA
Trim63	NM_001369245	TGTCTGGAGGTCGTTTCCG	ATGCCGGTCCATGATCACTT
Fbxo32	NM_026346	TCACAGCTCACATCCCTGAG	AGACTTGCCGACTCTTTGGA
IL-1β	NM_008361	TGACGTTCCCATTAGACAACTG	CCGTCTTTCATTACACAGGACA
IL-10	NM_010548	CAAGGAGCATTTGAATTCCC	GGCCTTGTAGACACCTTGGTC
TLR4	NM_021297	GCTTTCACCTCTGCCTTCAC	GAAACTGCCATGTTTGAGCA
TLR9	NM_031178	GAAAGCATCAACCACACCAA	ACAAGTCCACAAAGCGAAGG
Slc39a14	NM_144808	GCTGCTGCTATTTGGGTCTG	GACAAAGGGGACCAGAAAGC
Acsl4	NM_019477	CAATAGAGCAGAGTACCCTGAG	TAGAACCACTGGTGTACATGAC
Hmox1	NM_010442	GAGGTCAAGCACAGGGTGA	CAGGCCTCTGACGAAGTGA
Sat1	NM_001291865	CTGAAGGACATAGCATTGTTGG	TTCCATTCTGCTACCAAGAAGT
Atg12	NM_026217	GCCTCGGAACAGTTGTTTATTT	CAGTTTACCATCACTGCCAAAA
Atg16l1	NM_001205391	TGCGTGGAATGATAGTCAACTA	TCAATCACCAACTGAGCTAACT
Map1lc3b	NM_026160	CCACCAAGATCCCAGTGATTAT	TGATTATCTTGATGAGCTCGCT
Gabarapl1	NM_020590	TCCCTGATCTGGATAAGAGGAA	AAAGAAGAATAAGGCGTCCTCA
Sqstm1	NM_011018	GAACACAGCAAGCTCATCTTTC	AAAGTGTCCATGTTTCAGCTTC
PD-1	NM_008798	ATGACTTCCACATGAACATCCT	CTCCAGGATTCTCTCTGTTACC
PD-L1	NM_021893	TGAGCAAGTGATTCAGTTTGTG	CATTTCCCTTCAAAAGCTGGTC
NOX-2	XM_011397013	TGAATGCCAGAGTCGGGATT	CGAGTCACGGCCACATACA
SOD1	NM_011434	TATGGGGACAATACACAAGGCT	CGGGCCACCATGTTTCTTAGA

**Table 2:** Primary antibodies used for Western blot experiments.

Primary Antibodies	Description
Fbxo32	Rabbit polyclonal; 1:1000–1:2000; A3193; ABclonal, Wuhan, China
4-hydroxynonenal	Rabbit polyclonal; 1:3000; ab46545; Cambridge, UK
3-nitrotyrosine	Mouse monoclonal; 1:3000; ab61392; Cambridge, UK
TLR4	Rabbit polyclonal; 1:500–1:1000; GB11519; Sercicebio, Wuhan, China
ACSL-4	Mouse monoclonal; 1:100–1:1000; sc-365230; Santa Cruz; Dallas, TX, USA
Beta actin	Mouse monoclonal; 1:20000–1:100000; 66009-1-Ig; Proteintech; CHIX, USA

**Table 3:** Secondary antibodies used for Western blot experiments.

Secondary Antibodies	Description
Horseradish Peroxidase-conjugated Antibody	Goat Anti-Rabbit IgG; 1:3000; GB23204; Sercicebio, Wuhan, China
Horseradish Peroxidase-conjugated Antibody	Goat Anti-Mouse IgG; 1:3000; G1214; Sercicebio, Wuhan, China

## Data Availability

The data presented in this study are available in the article.
